# Analysis of the genomic homologous recombination in *Theilovirus *based on complete genomes

**DOI:** 10.1186/1743-422X-8-439

**Published:** 2011-09-17

**Authors:** Guangming Sun, Xiaodan Zhang, Maoli Yi, Shihe Shao, Wen Zhang

**Affiliations:** 1 The Fourth Affiliated Hospital of Jiangsu University, 20 Zhengdong Road, Zhenjiang, Jiangsu 212001, China; 2 School of Medical Science and Laboratory Medicine, Jiangsu University, 301 Xuefu Road, Zhenjiang, Jiangsu 212013, China; 3 Yantai Yuhuangding Hospital, 20 Yudong Road, Yantai, Shandong 264000, China

**Keywords:** Theilovirus, Recombination, Phylogenetic analysis

## Abstract

At present, *Theilovirus *is considered to comprise four distinct serotypes, including Theiler's murine encephalomyelitis virus, Vilyuisk human encephalomyelitis virus, Thera virus, and Saffold virus. So far, there is no systematical study that investigated the genomic recombination of *Theilovirus*. The present study performed the phylogenetic and recombination analysis of *Theilovirus *over the complete genomes. Seven potentially significant recombination events were identified. However, according to the strains information and references related to the recombinants and their parental strains, four of the recombination events might happen non-naturally. These results will provide valuable hints for future research on evolution and antigenic variability of Theilovirus.

## Introduction

*Encephalomyocarditis virus *(EMCV) and *Theilovirus *are two distinct species in the Cardiovirus genus of the family Picornaviridae [1]. The EMCVs comprise a single serotype and have a wide host range, while the *Theilovirus *species, probably includes four serotypes: Theiler's murine encephalomyelitis virus (TMEV), Vilyuisk human encephalomyelitis virus (VHEV), Thera virus (TRV; isolated from rats) and Saffold virus (SAFV; isolated from humans). TMEVs were originally isolated from mice and later from rats [2]. Serological studies indicated that the feral house mouse Mus musculus is the natural host for TMEV [3]. VHEV was isolated by the inoculation of mice with nasopharyngeal secretions, serum samples, feces, cerebrospinal fluid (CSF) specimens, and brain specimens from the Yakut-Evenk population, indigenous rural people in Siberia that had a chronic form of encephalitis [4]. TRV was isolated from sentinel rats housed with TMEV-seropositive rats in Japan [5]. This virus has not yet been associated with disease in rats but has raised the possibility of additional clades of undiscovered theiloviruses. SAFVs, new theiloviruses, were first isolated in California from a fecal sample from an 8-month-old infant with fever of undetermined origin [6] and then from a nasopharyngeal sample collected from a 23-month-old child in Canada in 2006 [7].

For picornaviruses, recombination is a common mechanism of evolution and antigenic variability. Although a recent report suggested that recombination happened in Cardiovirus genus [8], no study has systematically investigated the recombination among *Theilovirus *strains. In the present study, therefore, we systematically analyzed the available complete *Theilovirus *genome sequences in GenBank to elucidate the recombination among these viruses.

## Methods

### Sequences

The study sequences comprised all the 23 available complete genome sequences of *Theilovirus *from GenBank dated January 2011. Sequences were firstly screened to exclude patented and artificial mutants, and then aligned in the ClustalW program [9]. The alignment was manually adjusted for the correct reading frame. Sequences showing less than 1% divergence from each other were considered as the same. The strain information of the remaining 21 *Theilovirus *genomes were shown in Table 1. Because there was no complete genome of VFHV in GenBank before our analysis, this virus was not analyzed in the present study.

**Table 1:** The 21 *Theilovirus *strains used in phylogentic and recombination analysis in the present study

**GenBank No**.	Strain Name	Source	Country	Virus
M16020	BeAn	Mouse	USA	TMEV
M20301	DA	Mouse	USA	TMEV
X56019	GDVII	Mouse	UK	TMEV
EU718733	Vie415HRT	Mouse	USA	TMEV
EU723238	Yale	Mouse	USA	TMEV
EU718732	TOB15	Mouse	USA	TMEV
AB090161	NGS910	Rat	Japan	TRV
EU542581	TRV-1	Rat	USA	TRV
EU815052	RTV1	Rat	USA	TRV
EU681178	D/VI2273/2004	Human	Germany	SAFV
EU681179	D/VI2223/2004	Human	Germany	SAFV
FJ463616	Pak5152	Human	Pakistan	SAFV
FJ463615	Pak5003	Human	Pakistan	SAFV
FJ463617	Pak6572	Human	Pakistan	SAFV
EF165067	NA	Human	USA	SAFV
EU681177	BR/118/2006	Human	Germany	SAFV
EU681176	D/VI2229/2004	Human	Germany	SAFV
GU595289	HTCV-UC6	Human	USA	SAFV
EU376394	NA	Human	USA	SAFV
AM922293	Can112051-06	Human	Canada	SAFV
FM207487	Nijmegen2007	Human	Netherlands	SAFV

### Phylogenetic analysis

Before phylogenetic analysis, multiple-alignment was performed in the ClustalW program. Phylogenetic trees were constructed using the neighbor-joining method and evaluated using the interior branch test method with Mega 4 software [10]. Percent bootstrap support was indicated at each node. GenBank accession no. was indicated at each branch.

### Recombination Detection

The remaining 21 *Theilovirus *genomes were re-aligned in the ClustalW program. Detection of potential recombinant sequences, identification of potential parental sequences, and localization of possible recombination break points were determined using the Recombination Detection Program (RDP)[11], GENECONV [12], BOOTSCAN [13], MaxChi [14], CHIMAERA [15], and SISCAN [16] methods embedded in RDP3 [17]. A Multiple-comparison-corrected P-value cutoff of 0.05 was used throughout.

## Results and Discussion

**Figure 1:**
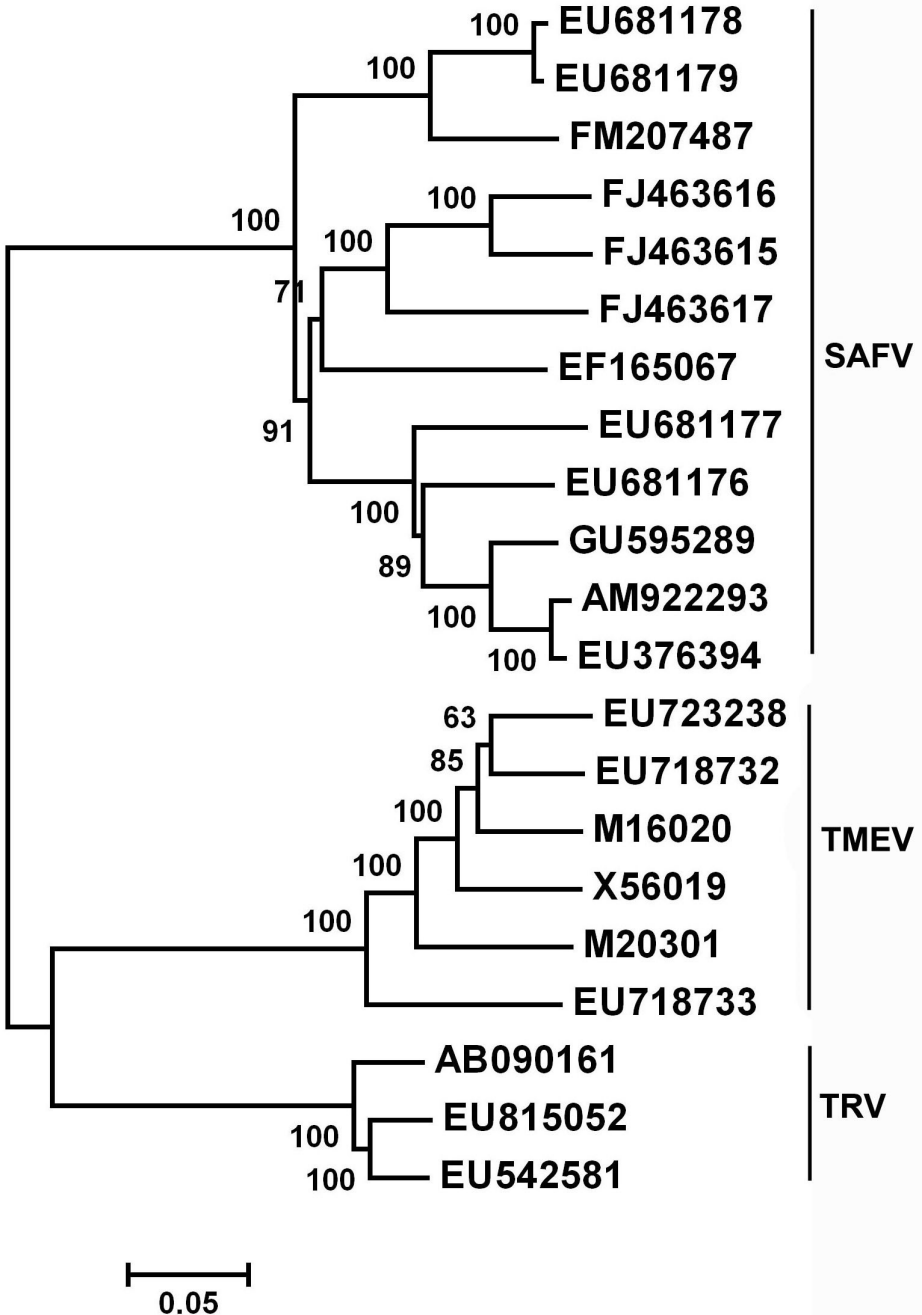
**Phylogenetic tree for the 21 complete *Theilovirus *genomes**. Phylogenetic analysis were performed using the neighbor-joining method and evaluated using the interior branch test method with Mega 4 software. Values for various branches are percentages of the tree obtained from 1000 resamplings of the data. Percent bootstrap supports are indicated at nodes.

Based on the 21 complete *Theilovirus *genomes, a phylogenetic tree was constructed (Figure 1). The taxonomy of these *Theilovirus *showed in the phylogenetic tree was consistent with the strain information from the original sources. From the phylogenetic tree, we can see that *Theilovirus *were divided into two major different genetical groups. Among the two major groups, SAFV formed a single group, while TMEV and TRV closely clustered, forming the other group. Sequence alignment indicated that TMEV strains shared 71.2%-75.3% and 67.4%-70.1% sequence identities with TRV and SAFV strains, respectively. While TRV strains showed 72.2%-74.8% sequence homologies to SAFV strain.

**Figure 2:**
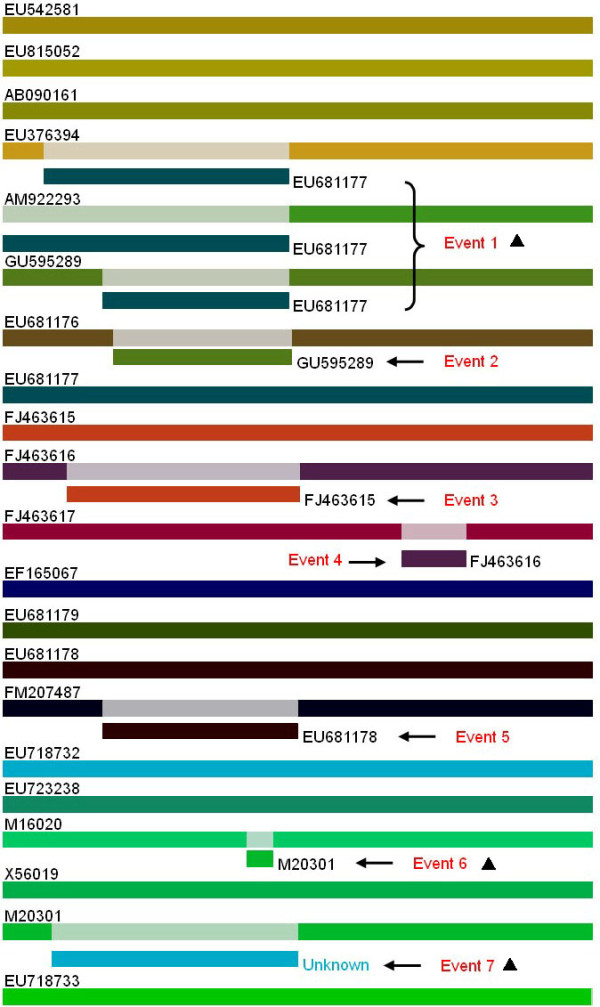
**Identification of the 7 recombination events**. The recombination events were indicated in red word "event"; GenBank No. of each strain was indicated at the left end; the minor parental strain of each recombinant was shown at the recombination region. The solid triangles indicated the naturally occurred recombination events.

Seven potentially significant recombination events were detected with a high degree of confidence (p value ≤ 1.3 × 10^-4^) judged by the above-mentioned six recombination detection methods. Figure 2 indicated the 7 recombination events, where we can see that event1 included three recombinants which had the same parental strains while the other six recombination events contained six recombinants, respectively.

**Figure 3:**
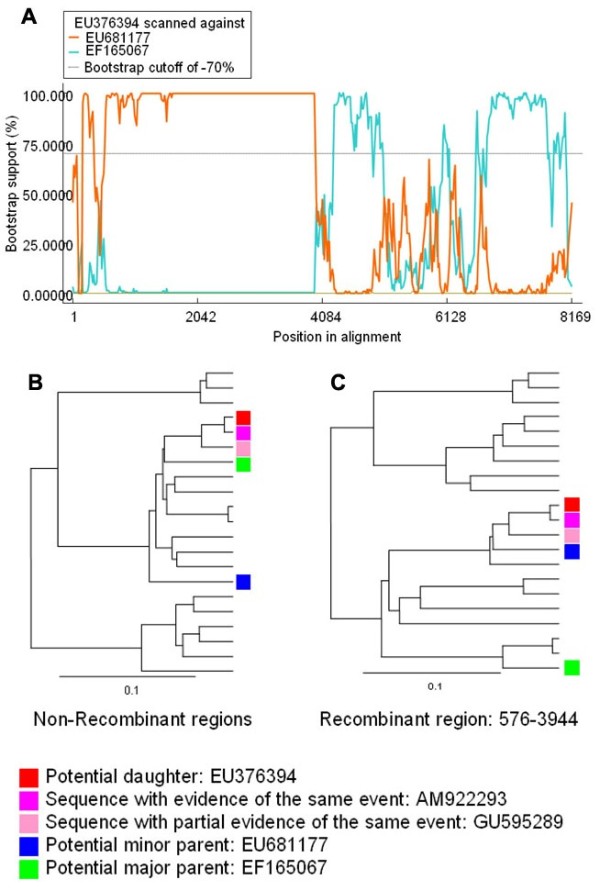
**Identification of recombination between EU681177 and EF165067**. (A) BOOTSCAN evidence for the recombination origin on the basis of pairwise distance, modeled with a window size 200, step size 20, and 100 Bootstrap replicates; (B) Neighbor joining tree (2,000 replicates, Kimura 2-parameter distance) constructed using the non-recombinant region (Position 1-575 + 3945-end); (C) Neighbor joining tree (2,000 replicates, Kimura 2-parameter distance) constructed using the recombinant region (Position 576-3944).

Figure 3 showed the identification result of recombination event1, which occurred between the lineage represented by a Germany SAFV strain [GenBank: EU681177] [18] as the minor parent and a USA SAFV strain [GenBank:EF165067] [6] as the major parent. This recombination event led to three recombinant SAFV strains [GenBank:EU376394, EMBL:AM922293, [GenBank:GU595289 ][7,19,20]. In this recombination event, the two parental strains were isolated in different countries, and the three daughter recombinants were distributed in different countries, which might hint that this recombination event happened long time ago and the recombinants were prevalent worldwide.

Recombination event2 identified the recombination occurred between two SAFV strains [GenBank:GU595289, GenBank:EU681179], leading to the other recombinant SAFV strain [GenBank:EU681176] (Additional File 1, Part A). However, in this recombination event, one of the parental strain [GenBank:EU681179] and the daughter strain were sequenced in the same lab [19], therefore, whether this recombination event occurred naturally or not should be verified by future studies. Additional File 1, Part B and C indicated the recombination event3 and event4, respectively, and three SAFV strains [GenBank:FJ463615, GenBank:FJ463616, GenBank:FJ463617] involved in the two recombination events were all sequenced in the same lab [21], therefore, it should be cared whether these two recombination events non-naturally occurred by sequencing error and/or contamination. The recombination event5 (Additional File 1, Part D) also contained two strains [GenBank:EU681179, GenBank: EU681178] which were isolated in the same lab [18], therefore, whether this recombination event non-naturally occurred by sequencing error and/or contamination should be elucidated by further study.

**Figure 4:**
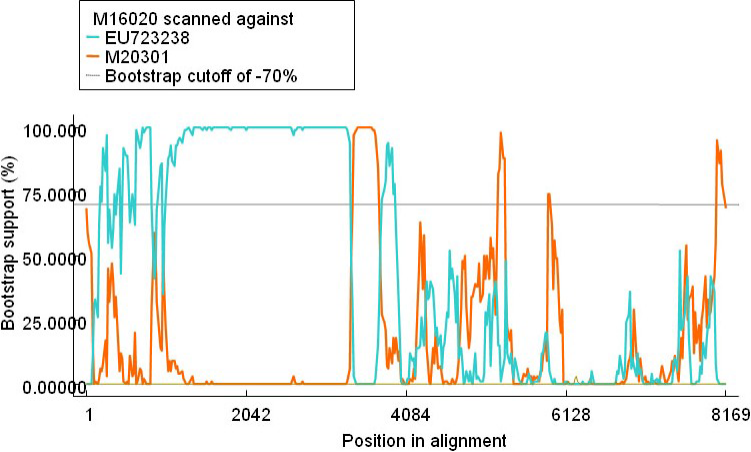
**BOOTSCAN evidence for recombination between two TMEV strains, which led to a recombinant TMEV strain**. Analysis were based on the basis of pairwise distance, modeled with a window size 200, step size 20, and 100 Bootstrap replicates.

**Figure 5:**
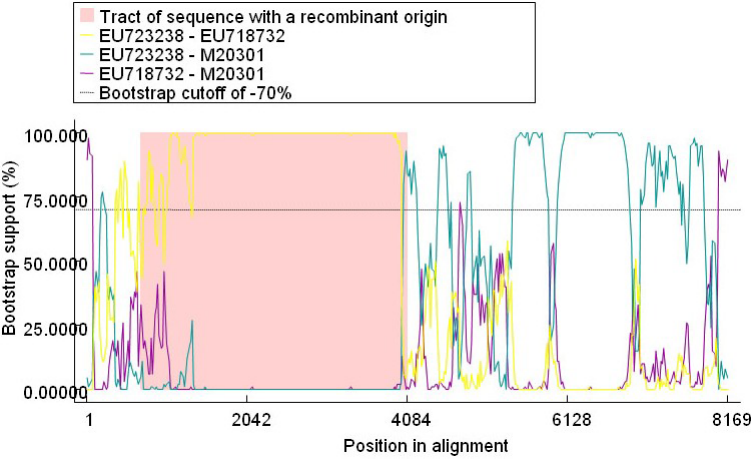
**RDP screenshots displaying the possible recombinant **(GenBank:M20301). The y-axis indicates the pairwise identity that refers to the average pairwise sequence identity within a 30nt sliding window moved one nucleotide at a time. The area outlined in gray demarcates the potential recombinant regions.

Figure 4 indicated the recombination event6 that occurred between a two TMEV strains, Yale strain [GenBank:EU723238] and DA strain [GenBank:M20301] [22], which led to the recombinant TMEV strain BeAn [GenBank:M16020] which was isolated from mouse in 1987, and these three virus strains were all isolated from mouse in USA [1,22]. Figure 5 revealed the putative recombinant TMEV strain (GenBank:M20301), however, the accurate parental strains has not been detected in the present study, which may due to the limited numbers of *Theilovirus *sequence available at present, therefore, further study should be performed to identify the accurate parental strains with the increasing number of *Theilovirus *genome sequences.

Recombination is a relatively common phenomenon in RNA viruses and understanding recombination will be helpful in unravelling the evolution of pathogens and drug resistance [23-25]. In the present study, we performed phylogenetic and recombination analyses over the full genome of *Theilovirus *available in GenBank nowadays. Seven potentially significant recombination events were detected. However, four of the recombination events might happen non-naturally in the lab, which should be taken into notice in the future evolutionary analysis of *Theilovirus*. The other three recombination events were further analyzed using other algorithms in RDP software bag and some of them were confirmed by phylogenetic analysis. The recombination phenomena of *Theilovirus *will also be noted in the further research because this will be one pattern of virulence factor variation in *Theilovirus*.

## Competing interests

The authors declare that they have no competing interests.

## Authors' contributions

GS conceived the study. All authors performed recombination analysis, critically reviewed, and approved the final manuscript. GS wrote the paper. All authors read and approved the final manuscript

## Supplementary Material

Additional file 1 **BOOTSCAN evidence for the recombination event 2, 3, 4, and 5**. Analysis was based on pairwise distance, modeled with a window size 200, step size 20, and 100 Bootstrap replicates. Click here for file
