# Supplementary figures and images for: Analysis of the genomic homologous recombination in Theilovirus based on complete genomes
Source: Virol J. 2011 Sep 17;8:439. doi: 10.1186/1743-422X-8-439 (PMC3183034; doi:10.1186/1743-422X-8-439)

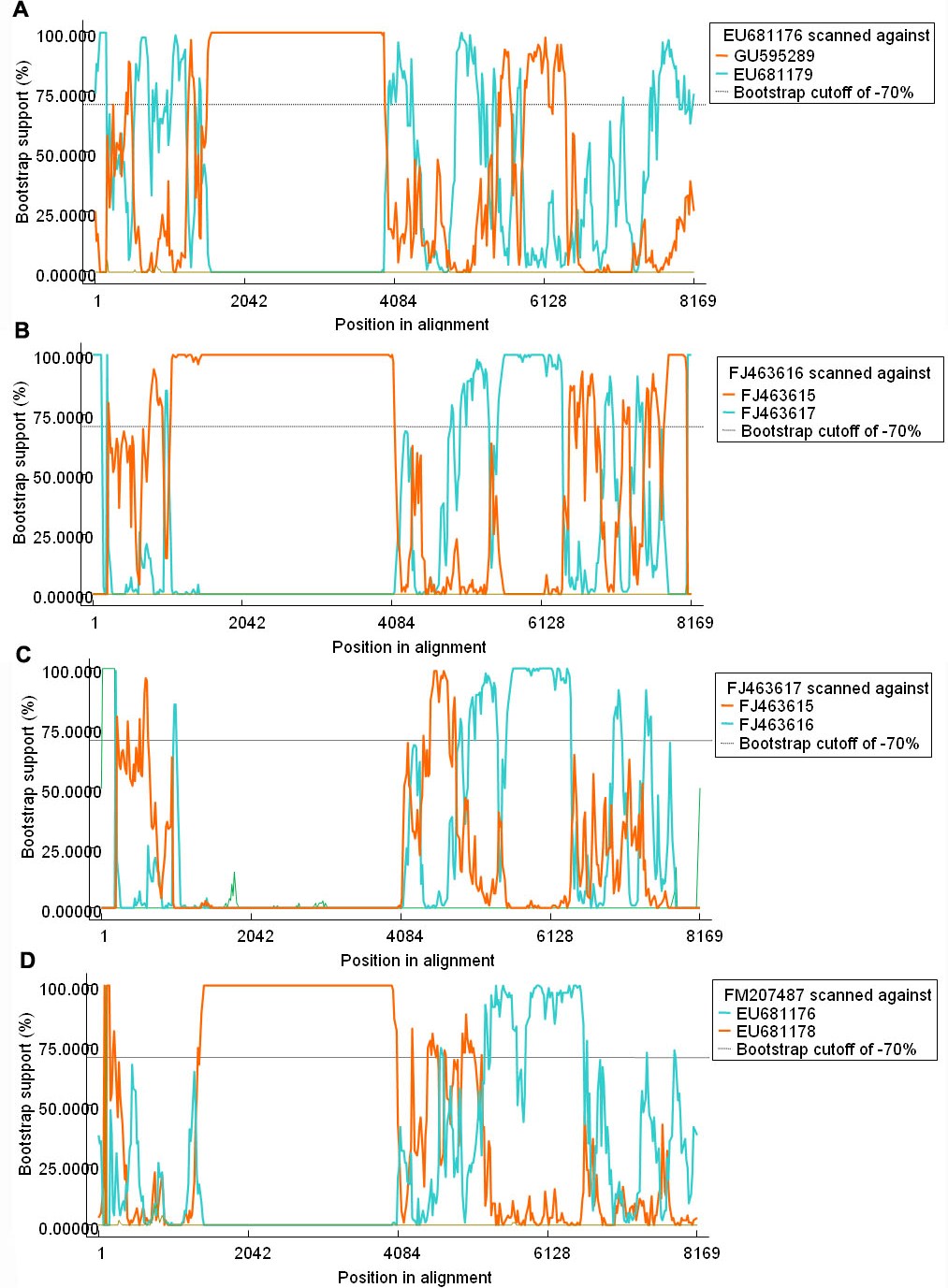

Supplement: Additional file 1 — BOOTSCAN evidence for the recombination event 2, 3, 4, and 5. Analysis was based on pairwise distance, modeled with a window size 200, step size 20, and 100 Bootstrap replicates. [file 1743-422X-8-439-S1.JPEG]
